# Glutamate potentiates susceptibility of *P. aeruginosa* clinical isolates to gentamycin: a new adjuvant hope for an old antibiotic

**DOI:** 10.3389/fmicb.2025.1677230

**Published:** 2026-01-14

**Authors:** Wedad M. Nageeb, Helal F. Hetta, Dan Frampton, Nigel J. Saunders

**Affiliations:** 1 Department of Medical Microbiology and Immunology, Faculty of Medicine, Suez Canal University, Ismailia, Egypt; 2 College of Health, Medicine and Life Sciences, Brunel University, London, United Kingdom; 3 Department of Medical Microbiology and Immunology, Faculty of Medicine, Assiut University, Assiut, Egypt; 4 Division of Microbiology, Immunology and Biotechnology, Department of Natural Products and Alternative Medicine, Faculty of Pharmacy, University of Tabuk, Tabuk, Saudi Arabia; 5 UCL Division of Infection and Immunity, University College London, London, United Kingdom; 6 Genpax, London, United Kingdom

**Keywords:** antibiotic uptake, gentamycin, glutamate, membrane permeability, *P. aeruginosa*

## Abstract

Aminoglycosides have long been considered essential agents in *P. aeruginosa* chemotherapy; however, resistance is on the rise. Glutamate is a crucial molecule for all living organisms, serving a fundamental function in various metabolic activities. A deeper comprehension of metabolic flux may facilitate effective therapeutic strategies for the eradication of antibiotic-resistant microorganisms. In this work, we aim to investigate a novel approach of amino acid-antibiotic adjuvant to potentiate activity of existing therapeutics through a low-toxicity adjuvant strategy offering lower risk of unwanted effects. We used 25 *P. aeruginosa* whole-genome sequenced clinical isolates selected from a genomically diverse set of isolates. The effect of glutamate-gentamycin combination on bacterial growth was tested. The effect of gentamycin-glutamate combination on outer membrane permeability was also studied using NPN. GWAS analysis was implemented using Pyseer to investigate genes underlying the observed enhanced effect of glutamate/gentamycin combination on bacterial killing. Among the tested 25 *P. aeruginosa* isolates, fifteen isolates demonstrated enhanced combinatorial effect of the gentamycin-glutamate combination resulting in enhanced bacterial killing effect. Interestingly observed, a set of genes involved in glycerophospholipid metabolism, purine metabolism, and glutamate metabolic pathways were interconnected showing significant differences among the two phenotypic groups using Pyseer. The uptake of fluorophore NPN in the presence of glutamate and two sub-inhibitory concentrations of gentamycin was increased indicating increased membrane permeabilization. This study reports the enhanced antibacterial effect of gentamycin-glutamate combination against *P. aeruginosa* opening doors for novel alternative treatment approaches against drug-resistant bacteria. Metabolic reprogramming offers a promising potential to enhance antibiotic mediated bacterial killing.

## 1 Introduction

Aminoglycosides were discovered via rigorous screening of soil Actinobacteria that commenced in the 1940s. Streptomycin, the first aminoglycoside, was isolated from *Streptomyces griseus* and was employed in the treatment of tuberculosis, subsequently addressing other infections caused by Gram-negative bacteria in 1944 (Schatz et al., 1944). Aminoglycosides agents have been widely used in medicine with growing use because of the increasing progress in developing effective derivatives since 1970s (Mingeot-Leclercq et al., 1999).

Aminoglycosides have historically been regarded as crucial drugs in the chemotherapy of *Pseudomonas aeruginosa*. They are used in the treatment of a wide variety of clinical infections including pulmonary infections, blood stream infections, urinary infections, eye, wound and burn infections (Nageeb and Hetta, 2022). Aminoglycosides are bactericidal agents and usually exhibit synergy, hence used in combination with other anti-*Pseudomonas* agents, most notably with beta-lactams (Edson and Terrell, 1999). They are widely recognized in the combinatorial therapy regimens for severe Gram-negative bacterial infections (Elfadadny et al., 2024).

Aminoglycosides facilitate their own uptake by interacting with bacterial lipopolysaccharides on the outer membrane of Gram-negative bacteria (Poole, 2005). The mechanism by which aminoglycoside antibiotics penetrate the Gram-negative bacterial cell wall is not fully understood; however, it is proposed to involve three distinct stages. The initial phase, as per the prevailing hypothesis, involves an electrostatic interaction between the positively charged aminoglycosides (AGAs) and the negatively charged lipopolysaccharides (LPS) of the outer bacterial membrane. The two subsequent stages are the energy dependent phase I (EDPI) which is characterized by a slow rate of uptake that is correlated with aminoglycosides concentrations and the energy-dependent phase II (EDPII) that uses energy from electron transport and ATP hydrolysis (Taber et al., 1987). The first influx of aminoglycoside molecules induces misreading during protein translation, compromising the integrity and function of the cytoplasmic membrane due to defective proteins, resulting in an autocatalytic cycle of AGA uptake and subsequent cell death (Davis et al., 1986). The outer membrane has been associated with aminoglycoside resistance, including resistance observed in clinical isolates (Hasegawa et al., 1997; Yoneyama et al., 1991; Shearer and Legakis, 1985). The observation is expected, given that LPS appears to be a critical target for aminoglycoside binding during its translocation across the outer membrane of *Pseudomonas aeruginosa*.

According to CDC national data for antimicrobial resistance in isolates from healthcare associated infections reported to the National Healthcare Safety Network, aminoglycoside resistance in *P. aeruginosa* in the USA was reported at the rate of 8.8% between 2011–2021 (782/8857 tested isolates) (National Healthcare Safety Network [NHSN], 2022). In Europe, rates of aminoglycoside resistance in *P. aeruginosa* were reported at 12.9% in 2018 and 8.9% in 2022 according to EARS-Net (ECDC, 2023). In China, the highest rate of aminoglycoside resistance in *P. aeruginosa* between 2019–2020 was reported at 8.2% according to CARSS.

There is a necessity for novel antibiotics and alternative treatment strategies to address drug-resistant bacteria. Metabolic modulation represents a promising novel approach that has been shown to enhance antibiotic efficacy. Exogenous metabolites have been documented to improve the efficacy of antibiotics against multidrug-resistant bacteria; however the underlying mechanisms remain mostly unidentified. Previous research reports have shown that an increased abundance of exogenous carbon source enhances bacterial inactivation by aminoglycoside antibiotics (Allison et al., 2011; Peng et al., 2015), thus, enhanced comprehension of metabolic flux could aid in the formulation of effective treatment or preventive strategies for the elimination of antibiotic-resistant bacteria.

Amino acids are essential biomolecules and the basic building components of proteins, with a burgeoning paradigm recognizing their potential as medicinal agents. The multimodal role of amino acids in microbial control has been recently recognized as potential antimicrobial agents, antibiofilm agents, drug excipients or drug-adjuvants (Idrees et al., 2020). There is evidence that the antibacterial activity of some drugs including ciprofloxacin, ceftazidime, imipenem, amikacin, and colistin (for Gram-negative bacteria) has been reported to have enhanced when combined (physically) with d-amino acids (Sanchez et al., 2017).

L-glutamate is the most important amino acid produced (3.3 million tons per year) by fermentation. L-Glutamic acid has become the predominant segment of amino acid products, representing over 40% of the entire market volume in 2014 (Sanchez et al., 2017). Glutamate is an important molecule for all living organisms playing a central role in a wide range of metabolic processes in bacterial cells. It is a non-essential amino acid that serves as a key metabolite acting as a link between nitrogen and carbon metabolism (Berg and John, 2002). It is regarded as a significant metabolic center in numerous organisms and is thus engaged in other processes beyond protein synthesis. Very minimal data is available in the literature about the microbial toxicity of glutamate. A study has previously demonstrated a minimal toxic effect of monosodium glutamate against *Pseudomonas aeruginosa* (MTCC 424) with the reference strain being inhibited only at high concentrations of glutamate (Bhuvaneswari et al., 2015). More recently, monosodium glutamate used as a food additive has also shown mild inhibitory effect against some Gram-negative pathogens (Diaaeldin et al., 2022).

This study investigates the novel use of monosodium glutamate as a drug adjuvant, extending its conventional function as a food additive. We aim to investigate a novel approach of amino acid-antibiotic adjuvant to potentiate activity of existing therapeutics through a low-toxicity adjuvant strategy offering lower risk or unwanted effects.

## 2 Materials and methods

### 2.1 Study isolates

Eighty-seven highly diverse isolates of *P. aeruginosa* were selected from an in-house collection developed at Brunel University London. Strains included in the study were kindly provided by Prof. David Livermore (Livermore et al., 1981) and from the BSAC Bacteremia Resistance Surveillance Program (Reynolds et al., 2008). Antibiotic sensitivity testing was performed using the Oxoid M.I.C. Evaluator (Thermo Fisher Scientific) to measure MIC values according to the manufacturer’s instructions.

### 2.2 Whole genome DNA extraction, sequencing and assembly

Whole genomic DNA extraction for *P. aeruginosa* isolates was done using the FastDNA^®^ SPIN Kit and the FastPrep^®^ Instrument (MP Biomedicals, Santa Ana, CA). Isolates were sequenced in Wellcome Trust Centre for Human Genetics using Illumina next generation 150-bp paired-end sequencing with 192 multiplexed libraries protocol to generate 14–45× coverage on Illumina HiSeq2500. Each genome was assembled using the *de novo* sequence assembly program SPAdes (Bankevich et al., 2012). Assembly quality and downstream sequence analyses were carried out using MUMmer (Delcher, 2002), BLAST (Altschul et al., 1990), and in-house perl scripts. Read files are submitted to the ENA (European Nucleotide Archive) with study accession number: PRJEB31646.

### 2.3 Phenotypic context of studied isolates

We have experimentally tested 25 *P. aeruginosa* isolates out of a total of 87 isolates which were completely sequenced and selected for diversity. The isolates tested included different gentamycin phenotypes including 3 isolates with MIC of 32, 4 isolates with MIC of 16, 10 isolates with MIC of 8, and 8 isolates with MIC of 4. Each isolate was tested at 2 different sub-MIC concentrations of gentamycin in 2 biologic replicates and 3 technical replicates each.

### 2.4 Assessment of the genomic context of studied *P. aeruginosa* isolates

The isolates used in this study were chosen as a set of strains best representing species diversity from a larger collection of isolates developed at Brunel University London. Strains with greater than 13,500 SNPs in the core genome separating any strain from its nearest neighbor in the collection were selected for use in this study.

The CSI phylogeny tool by the DTU available at https://cge.food.dtu.dk/services/CSIPhylogeny/ was used to constructs whole genome SNP-based dendrogram (Kaas et al., 2014). SAMtools (Li et al., 2009) were used for sequence alignment and mapping and MuMmer (Delcher et al., 2002) for large scale genome alignment and comparison. Concatenated alignment of high-quality SNPs was then used to infer phylogeny. The minimum depth at SNP position used was 10× and the minimum distance between SNPs was 10 bp. Approximate maximum-likelihood for large alignments as implemented in Fast tree (Price et al., 2010) was used to infer phylogeny.

### 2.5 Investigating genetic relatedness of tested isolates

Investigating genomic background of the experimentally tested isolates was performed through comparing the most important genes related to glutamate uptake and utilization in *P. aeruginosa* (Lundgren et al., 2021) including *aat*J (PAO1: PA1342), aatQ (PAO1: PA1341), *aat*M (PAO1: PA1340), *aat*P (PAO1: PA1339), *aau*R (PAO1: PA1335) and *rpo*N (PAO1: PA4462) and those membrane related genes with previously reported role in aminoglycosides uptake or resistance including *opr*H (PAO1: PA1178), *nuo*G (PAO1: PA2642), *rpl*Y (PAO1: PA4671), *gal*U (PAO1: PA2023), *pst*B (PAO1: PA5366), *fao*A (PAO1: PA3014), *lpt*A (PAO1: PA0005), *arn*A (PAO1: PA3554), *arn*B (PAO1: PA3552), *arn*C (PAO1: PA3553) and *arn*D (PAO1: PA3555).

The BLAST analysis for the genes in question was done using NCBI BLAST+ blastn (Version 2.10.0) integrated in Galaxy (Camacho et al., 2009; Cock et al., 2015). The BLAST outputs for each gene for all tested isolates were downloaded and aligned using mega software (Tamura et al., 2021).

Sequence matrix tool (Vaidya et al., 2011) was used to concatenate aligned gene sequences from the studied 25 isolates into a super matrix. The multilocus super matrix including the mentioned genes was used to infer genetic relatedness and phylogeny of studied strains.

The analysis was performed by combining the mentioned genes in three sets. The first concatenated set included membrane genes with a role in aminoglycoside resistance. The second set included genes with a role in glutamate uptake and the third analysis set included concatenating both the previous sets together.

Maximum-likelihood phylogenetic trees were generated from each alignment using IQTREE2 (Minh et al., 2020) with support values obtained via 1,000 ultrafast bootstraps (Hoang et al., 2018). For each alignment, ModelFinder (Kalyaanamoorthy et al., 2017) was used to identify and apply the substitution model with the lowest Bayesian information criterion (BIC).

### 2.6 Testing the additive effect of glutamate and gentamycin combination on bacterial growth

The starting bacterial pre-culture was inoculated into an 8 ml Cation Adjusted Muller-Hinton broth (CAMHB) from an overnight culture of fresh colonies and incubated overnight at 37°C to reach the stationary phase of growth. Experimental bacterial suspension of tested isolates was grown from an overnight pre-culture (at the stationary phase) into a well aerated 30 ml tube (1 ml of pre-culture is added to 15 ml CAMHB) to reach early log phase. Cultures were incubated in a shaking incubator at 37.5 °C for 1.5–3 h with shaking at 150 rpm. OD_620_ of isolates was measured and adjusted to 0.1 to 0.2. The suspension obtained from this growth containing 10^7^–10^8^ CFU/ml was then used as the inoculum for the microplate reader growth experiment. Antibiotic solutions to be tested were prepared at 1/3 and 1/6 of the MIC for each tested isolate and glutamate solutions were also prepared at the tested concentrations of 2 mM. Different concentrations were initially tried and then re-tested based on initial observations.

Microplates (96 well, Greiner Cat No. 655185) were used to carry out the experiment in SPECTROstar^®^ Nano instrument. Plates were sealed using gas-permeable BreatheEasy membranes (Sigma, Cat No. Z380059). Plates were then incubated at 37°C and shaken for 15 s prior to each OD reading. Absorbance measurements at OD_620_ were taken at intervals of 10 min for a total of 20–22 h. The measurements at these data points were used to draw a growth curve for each isolate under each tested condition. The growth rate was then calculated based on the maximum of slope (rising) in the full range of measured data points based on blank corrected values using SPECROstar^®^ Nano MARS Data Analysis Software which was also used to measure area under the curve for the full kinetic range of the growth curve and to analyze the results.

### 2.7 Genome wide association study (GWAS) using Pyseer

The 25 phenotypically tested isolates for the effect of glutamate gentamycin combination were divided into 2 sets. The first set showed an enhanced glutamate-gentamycin combinatorial effect and the second set showed no enhanced effect. Both sets were then compared using Pyseer for performing GWAS study (Lees et al., 2018) available at https://github.com/mgalardini/pyseer.

To investigate the possible functional roles of the output genes and their relation to the studied phenotype, the genes were mapped to their corresponding pathways and investigated for possible links and relations using the STITCH 5 database (Szklarczyk et al., 2016). Network visualization and analysis were performed using Cytoscape (Killcoyne et al., 2009).

### 2.8 Testing the effect of gentamycin-glutamate combination on permeabilization of the outer membrane to NPN

NPN is a hydrophobic probe, and its fluorescence is associated with its presence in a glycerophospholipid environment such as the lipid bilayer of biological membranes. Increased fluorescence values indicate weakening of the outer membrane. Wild type cells take up little or no NPN while enhancement of NPN uptake as measured by change in NPN fluorescence owing to NPN partitioning into the hydrophobic membrane interior is a direct measurement of increased outer membrane permeability (Hancock and Wong, 1984). NPN is a neutral probe which was chosen to avoid charge neutralization and to enable kinetic analysis. This probe fluoresces weakly in aqueous environment but very strongly in non-polar or hydrophobic environment (Loh et al., 1984).

NPN uptake by bacterial suspensions tested was measured using Thermo Scientific™ Nunc™ F96 MicroWell™ Black Microtitre Plate (Cat.No.10000631) in CLARIOstar^®^ instrument (excitation, 350 nm; emission, 460 nm). Experimental cultures were grown from an overnight CAMHB culture at 37.5 °C with vigorous shaking at 250 rpm for 2–3 h to an OD_620_ ∼ 0.5. Bacterial cultures (OD_620_ = 0.4–0.6) were collected by centrifugation at 3,500 rpm for 10–15 min and resuspended in an equal volume of (5 mM HEPES buffer). Cells were then diluted 1:100 in fresh broth. Aliquots (50 μl) of this cell suspension were added to the microplate containing 100 μl of gentamycin at both (0.125 μg/ml, 0.0625 μg/ml) final concentrations separately, glutamate (2 mM) final concentration separately or combination of both. Blank media was used as a control. NPN (10 μM) final concentration was then added immediately before measurement to a total volume of 200 μl. Cultures were then allowed to grow at 30 °C and 20% O_2_ with orbital shaking at 300 rpm. Fluorescence was monitored and continuously recorded every 4 min for 4 h. Fluorescence of the probe with bacterial cells suspensions incubated without adding any treatment as well as background fluorescence of the broth used was also measured and taken into account when calculating the net change in fluorescence. 3–6 wells were included per condition and each experiment was repeated twice.

## 3 Results

### 3.1 Description of the genetic background and phylogenetic context of studied *P. aeruginosa* isolates

**FIGURE 1:**
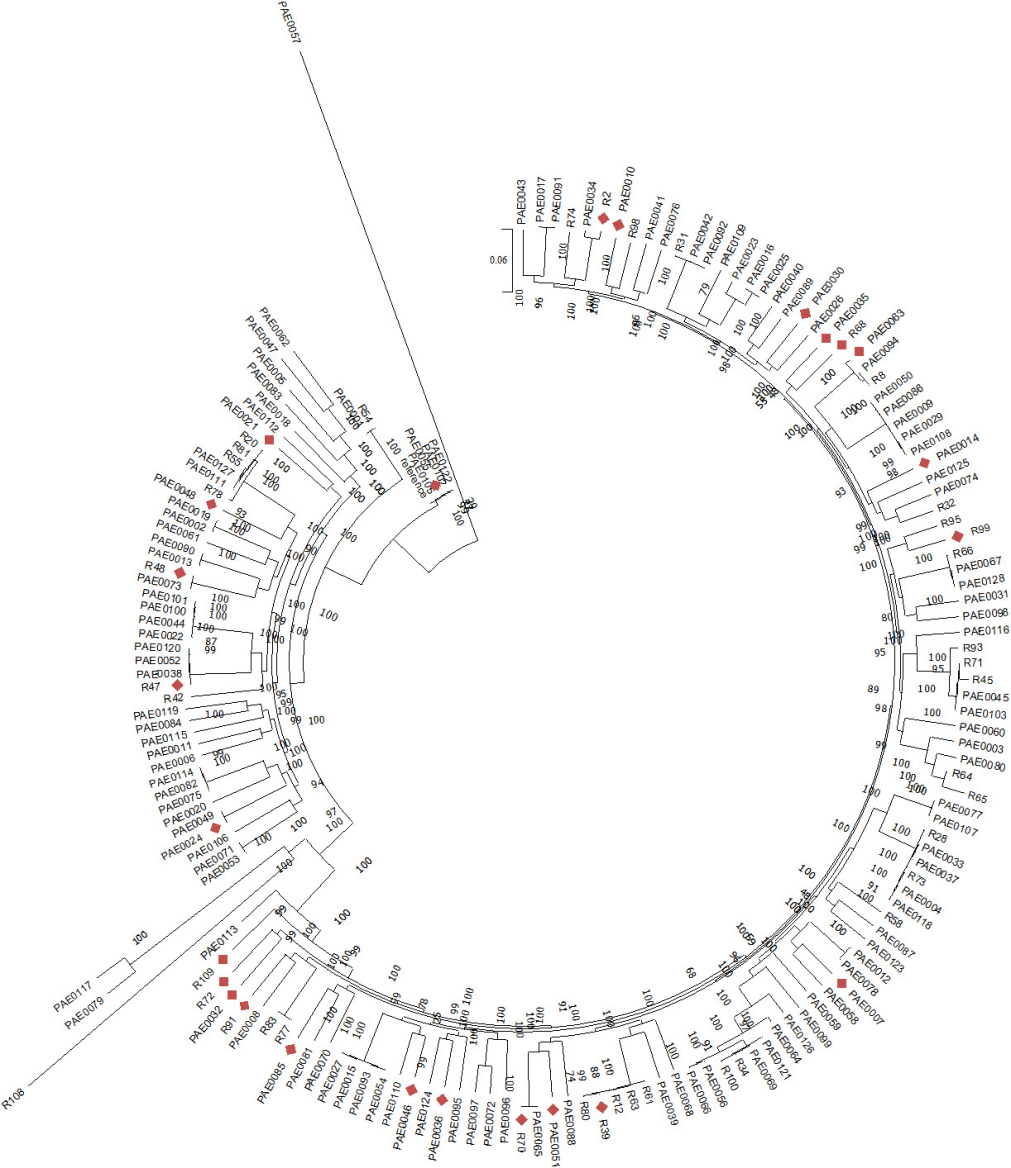
Whole genome-based phylogenetic tree showing the distribution of *P. aeruginosa* study isolates among a selected diverse collection. Red diamonds point to phenotypically tested isolates among the whole diverse set.

**FIGURE 2:**
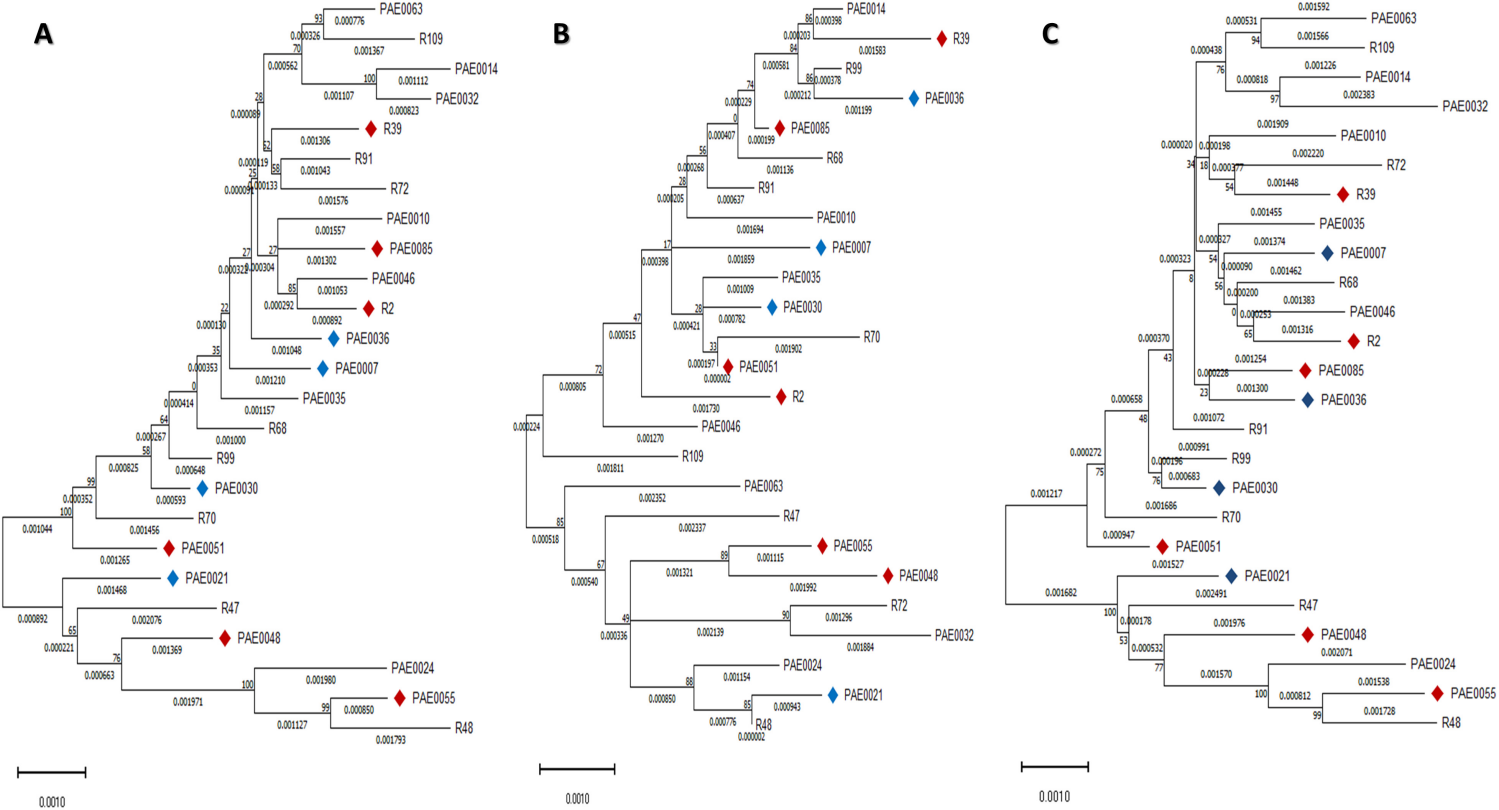
Genetic relatedness phylograms based on different concatenated genes trees. **(A)** Phylogeny based on concatenated set of genes related to aminoglycoside uptake across the cell membrane. **(B)** Phylogeny based on concatenated set of genes related to glutamate uptake and utilization. **(C)** Phylogeny based on concatenated sets of genes related to both aminoglycoside uptake and glutamate uptake. Isolates not showing enhanced glutamate/gentamycin effect are labeled with diamonds. Blue diamonds refer to isolates with MIC of 4. Red diamonds refer to isolates with MIC of 8.

Phylogenetic relationships of the most diverse collection of studied isolates are shown in Figure 1 showing the distribution of the 25 phenotypically tested isolates which were distributed across the tree. To infer relatedness of studied isolates, and to interpret for differences in the measured phenotype, background genomic phylogeny was constructed using a set of genes related to aminoglycoside uptake across the cell membrane and genes related to glutamate uptake and utilization. The variations in these genes were tested three times as three separate concatenated sets and were used to classify the tested isolates based on the observed behavior of enhanced glutamate gentamycin susceptibility to find the possible role of variations in these genes in determining the type of combined aminoglycoside and glutamate effect. Phylogenetic relatedness trees are shown in Figures 2A–C. Neither of the three classifications were able to separate the tested set into completely separate clusters based on variations in these genes.

### 3.2 Effect of gentamycin-glutamate combination on studied isolates

**FIGURE 3:**
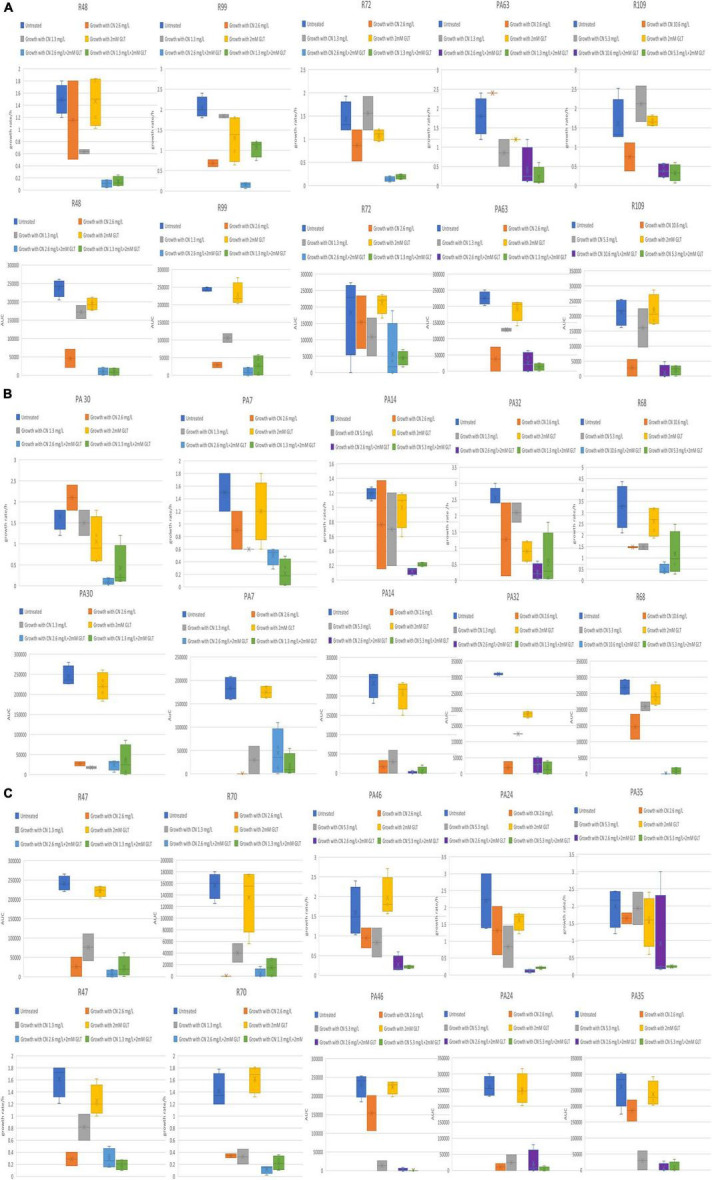
**(A–C)** Tested *P. aeruginosa* isolates showing enhanced combinatorial effect in growth rate and AUC with glutamate/gentamycin combination. CN, gentamycin; GLT, glutamate; AUC, area under the curve; growth rate/h, growth rate per hour.

**FIGURE 4:**
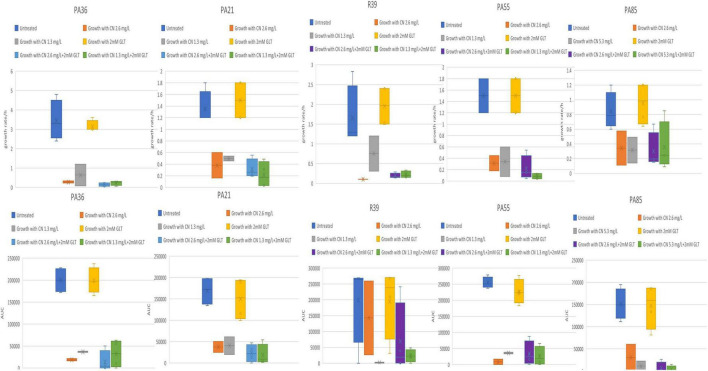
Tested *P. aeruginosa* isolates showing minimal or no observable differences in growth rate and AUC with glutamate/gentamycin combination. CN, gentamycin; GLT, glutamate; AUC, area under the curve; growth rate/h, growth rate per hour.

**FIGURE 5:**
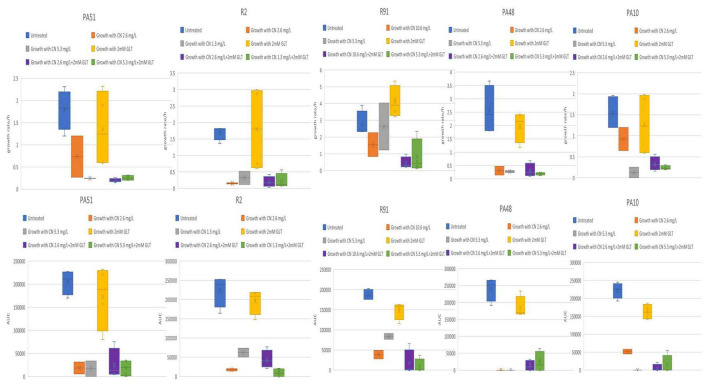
Tested *P. aeruginosa* isolates showing minimal or no observable differences in growth rate and AUC with glutamate/gentamycin combination. CN, gentamycin; GLT, glutamate; AUC, area under the curve; growth rate/h, growth rate per hour.

Among the 25 tested isolates, fifteen isolates demonstrated enhanced combinatorial effect of the gentamycin-glutamate combination as evidenced by the differences in growth rate and AUC when combination was used compared to each individual agent (Figures 3A–C). The other ten *P. aeruginosa* isolates did not demonstrate enhanced combinatorial effect as evidenced by absence of observable differences in growth rate and AUC when combination was used compared to each individual agent (Figures 4, 5).

### 3.3 Results of genome wide association study (GWAS) using Pyseer

To interpret the differences observed in glutamate-gentamycin combinatorial effect, GWAS was performed to find genes that may underlie the positive combinatorial effect of glutamate in increasing gentamycin susceptibility in *P. aeruginosa*.

GWAS output genes showing significant differences among the two groups (*P* < 0.05) and those showing the highest effect sizes (evidenced by beta value), excluding those undefined genes with unknown function and hypothetical proteins, are shown in Supplementary Table 1.

**FIGURE 6:**
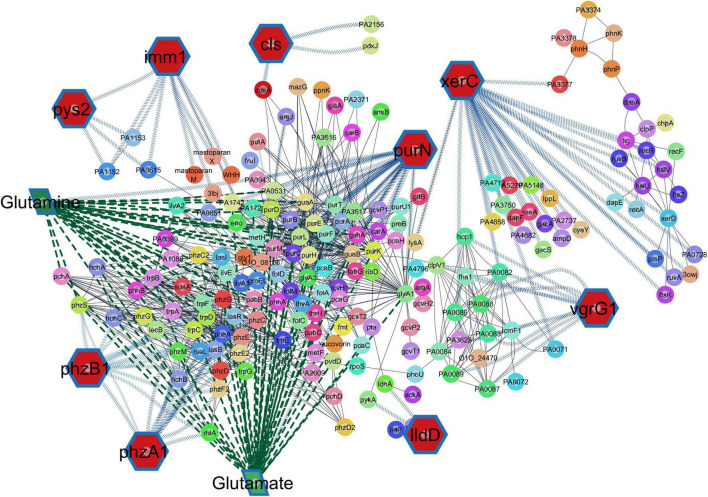
Network of output genes from GWAS results and interacting genes from the STITCH 5 database. Hub genes shown in red hexagons are those showing significant differences and highest effect sizes from Pyseer output analysis.

To investigate the possible functional roles of these genes and their relation to the studied phenotype, the genes were mapped to their corresponding pathways and investigated for possible links and relations using the STITCH 5 database. Output genes interactions and their corresponding pathways are shown in Table 1. Table 2 shows the predicted strength of interactions of different pairs of genes in the wider network of genes connecting the different pathways of fatty acid and phospholipid metabolism, nucleotide biosynthesis and metabolism, amino acid biosynthesis and metabolism, and protection and adaptation. Figure 6 shows the network of Pyseer output genes linked to other co-interacting genes as exported from the STITCH 5 database interactions and visualized using Cytoscape.

**TABLE 1:** Functional classification of the set of main interacting genes resulting from GWAS output and their closely related functional partners as mapped to corresponding pathways (From the *Pseudomonas* Genome database) (Winsor et al., 2016).

Gene	Function	Gene ontology biological process	Gene ontology molecular function	Pathways	Functional classifications manually assigned by PseudoCAP
*cls* [PA5394]	Cardiolipin synthetase; Catalyzes the reversible transfer of a phosphatidyl group between phosphatidylglycerol molecules, resulting in the formation of cardiolipin (CL) (diphosphatidylglycerol) and glycerol (By similarity)	Cardiolipin biosynthetic process	Catalytic activity Cardiolipin synthase activity Phosphotransferase activity, for other substituted phosphate groups	Metabolic pathways Glycerophospholipid metabolism Superpathway of phospholipid biosynthesis I (bacteria) Cardiolipin biosynthesis I	Fatty acid and phospholipid metabolism
*cds*A [PA3651]	Phosphatidate cytidylyltransferase	Cellular lipid metabolic process	Phosphatidate cytidylyltransferase activity	Metabolic pathways Glycerophospholipid metabolism Superpathway of phospholipid biosynthesis I (bacteria) CDP-diacylglycerol biosynthesis I–II Biosynthesis of secondary metabolites	Fatty acid and phospholipid metabolism
*pur*N *[PA0944]*	Phosphoribosylglycinamide formyltransferase. Phosphoribosylaminoimidazole synthetase; Facilitates the transfer of a formyl group from 10-formyltetrahydrofolate to 5-phospho-ribosyl-glycinamide (GAR), yielding tetrahydrofolate and 5-phospho-ribosyl-N-formylglycinamide (FGAR).	Biosynthetic process “*de novo*” IMP biosynthetic process Nucleotide metabolic process	Phosphoribosylglycinamide formyltransferase activity Succinate dehydrogenase activity	Purine metabolism One carbon pool by folate Superpathway of purine nucleotides *de novo* biosynthesis II Superpathway of histidine, purine, and pyrimidine biosynthesis Purine metabolism Metabolic pathways Biosynthesis of secondary metabolites 5-aminoimidazole ribonucleotide biosynthesis II Biosynthesis of antibiotics	Nucleotide biosynthesis and metabolism
*pur*D *[PA4855]*	Phosphoribosylamine–glycine ligase	Purine nucleobase biosynthetic process Nucleotide metabolic process Purine nucleotide metabolic process	Phosphoribosylamine-glycine ligase activity ATP binding UDP-N-acetylmuramoylalanine-D-glutamate ligase activity	Purine metabolism Superpathway of histidine, purine, and pyrimidine biosynthesis Superpathway of purine nucleotides *de novo* biosynthesis II Metabolic pathways Biosynthesis of secondary metabolites Biosynthesis of antibiotics 5-aminoimidazole ribonucleotide biosynthesis II	Nucleotide biosynthesis and metabolism
*pur*A *[PA4938]*	Adenylosuccinate synthetase; Plays a crucial function in the *de novo* process of purine nucleotide synthesis. Facilitates the initial committed step in the biosynthesis of AMP from IMP (By similarity) and is a member of the adenylosuccinate synthetase family.	Purine nucleotide biosynthetic process Cellular amino acid metabolic process Alanine metabolic process Aspartate metabolic process Nucleotide metabolic process	Biotin-[acetyl-CoA-carboxylase] ligase activity Adenylosuccinate synthase activity Nucleotide binding	Metabolic pathways Purine metabolism Alanine, aspartate and glutamate metabolism Adenosine ribonucleotides *de novo* biosynthesis	Nucleotide biosynthesis and metabolism Amino acid biosynthesis and metabolism
*gua*A *[PA3769]*	GMP synthase; Catalyzes the synthesis of GMP from XMP (By similarity)	GMP biosynthetic process Nucleotide metabolic process Cellular amino acid metabolic process Purine nucleotide biosynthetic process	GMP synthase (glutamine-hydrolyzing) activity ATP binding	Metabolic pathways Purine metabolism Glutamate metabolism	Nucleotide biosynthesis and metabolism Amino acid biosynthesis and metabolism
*arg*J [PA4402]	Glutamate N-acetyltransferase; Bifunctional ornithine acetyltransferase/N-acetylglutamate synthase catalyzes two activities integral to the cyclic pathway of arginine biosynthesis: the synthesis of N-acetylglutamate from glutamate and acetyl-CoA as the acetyl donor, and the production of ornithine via transacetylation between glutamate and N(2)-acetylornithine (By similarity).	Cellular amino acid metabolic process Arginine biosynthetic process	NADH dehydrogenase (ubiquinone) activity Glutamate N-acetyltransferase activity	Metabolic pathways L-arginine biosynthesis II (acetyl cycle) Biosynthesis of secondary metabolites Biosynthesis of antibiotics Biosynthesis of amino acids 2-Oxocarboxylic acid metabolism	Amino acid biosynthesis and metabolism
PA0531 [PA0531]	Glutamine amidotransferase			Guanosine ribonucleotides *de novo* biosynthesis	Putative enzymes
O1O_08108 *[PA2444]*	Serine hydroxymethyltransferase, Facilitates the reversible transformation of serine and glycine, utilizing tetrahydrofolate (THF) as the one-carbon transporter. This process is the primary source of one-carbon units necessary for the production of purines, thymidylate, methionine, and other essential biomolecules. Additionally demonstrates THF-independent aldolase activity toward beta-hydroxyamino acids, yielding aldehydes and glycine by a retro-aldol mechanism (By similarity)	Cellular amino acid metabolic process Threonine metabolic process Glycine metabolic process Methane metabolic process L-serine metabolic process Lysine catabolic process Glycine biosynthetic process from serine Tetrahydrofolate interconversion	3-methyl-2-oxobutanoate dehydrogenase (2-methylpropanoyl-transferring) activity Pyridoxal phosphate binding Glycine hydroxymethyltransferase activity	Superpathway of L-serine and glycine biosynthesis I Serine-isocitrate lyase pathway One carbon pool by folate N-formyl-tetrahydrofolate biosynthesis Microbial metabolism in diverse environments Methane metabolism Lysine degradation Glyoxylate and dicarboxylate metabolism Glycine, serine and threonine metabolism Cyanoamino acid metabolism Biosynthesis of secondary metabolites Biosynthesis of antibiotics Biosynthesis of amino acids	Amino acid biosynthesis and metabolism
imm1 *[PA1151]*	Pyocin S2 immunity protein These immunity proteins can bind specifically to the DNase-type colicins and pyocins and inhibit their bactericidal activity	Bacteriocin immunity	Toxic substance binding		Adaptation, protection
*pys*2 *[PA1150]*	Pyocin S2; Causes breakdown of chromosomal DNA as well as complete inhibition of lipid synthesis in sensitive cells	Cytolysis Response to bacterium	signaling receptor binding Endonuclease activity		Adaptation, protection Secreted Factors (toxins, enzymes, alginate)
*gua*B [PA3770]	Inosine-5′-monophosphate dehydrogenase; Facilitates the transformation of inosine 5′-phosphate (IMP) into xanthosine 5′-phosphate (XMP), the initial committed and rate-limiting phase in the *de novo* production of guanine nucleotides, hence significantly influencing cell growth regulation.	Purine nucleotide biosynthetic process Nucleotide metabolic process	4-hydroxybenzoate 3-monooxygenase activity IMP dehydrogenase activity Catalytic activity	Metabolic pathways Purine metabolism Urate biosynthesis/inosine 5′-phosphate degradation Guanosine ribonucleotides *de novo* biosynthesis Biosynthesis of secondary metabolites	Nucleotide biosynthesis and metabolism
ClpV1[PA0090]	ClpV1; Required for secretion of Hcp1 probably by providing the energy source for its translocation; Belongs to the ClpA/ClpB family.		ATP binding ATPase activity	Bacterial secretion system Biofilm formation–*Pseudomonas aeruginosa*	Protein secretion/export apparatus Translation, post-translational modification, degradation Chaperones and heat shock proteins
VgrG1[PA0091]	VgrG1; Part of the H1 type VI secretion system (H1-T6SS) unique secretion mechanism that translocates several virulence components into both prokaryotic and eukaryotic cells during infection. Constitutes the spike at the apex of the extending tube created by haemolysin co-regulated protein 1/Hcp1. Facilitates the transport of the Tse6 toxin to target cells, where it manifests its lethal effects.	Protein secretion by the type VI secretion system	Type VI protein secretion system complex	Bacterial secretion system	Protein secretion/export apparatus
XerC[PA5280]	Site-specific recombinase Sss; Site-specific tyrosine recombinase. It functions by catalyzing the cleavage and rejoining of the recombining DNA strands. The XerC-XerD complex is crucial for transforming dimers of the bacterial chromosome into monomers to facilitate their segregation during cell division. It also enhances the segregational stability of plasmids (by analogy). Is a member of the “phage” integrase family. XerC subfamily.	Cellular response to zinc ion DNA integration DNA recombination Cell division Chromosome segregation	Site-specific recombinase activity DNA binding		DNA replication, recombination, modification and repair
*lys*A [PA5277]	Diaminopimelate decarboxylase; Specifically catalyzes the decarboxylation of meso- diaminopimelate (meso-DAP) to L-lysine.	Cellular amino acid metabolic process Lysine biosynthetic process via diaminopimelate	Diaminopimelate decarboxylase activity Catalytic activity	Superpathway of L-lysine, L-threonine and L-methionine biosynthesis I Microbial metabolism in diverse environments Biosynthesis of secondary metabolites Biosynthesis of antibiotics	Amino acid biosynthesis and metabolism
*gly*A1[PA5415]	Serine hydroxymethyltransferase; Facilitates the reversible interconversion of serine and glycine, utilizing tetrahydrofolate (THF) as the one-carbon transporter. This process is the primary source of one-carbon units necessary for the production of purines, thymidylate, methionine, and other essential biomolecules. Additionally demonstrates THF-independent aldolase activity with beta-hydroxyamino acids, yielding glycine and aldehydes by a retro-aldol mechanism.	Cellular amino acid metabolic process Tetrahydrofolate interconversion Glycine biosynthetic process from serine	Glycine hydroxymethyltransferase activity Pyridoxal phosphate binding	Superpathway of L-serine and glycine biosynthesis I Serine-isocitrate lyase pathway One carbon pool by folate N- formyl-tetrahydrofolate biosynthesis Microbial metabolism in diverse environments Lysine biosynthesis Glycine, serine and threonine metabolism Cyanoamino acid metabolism Biosynthesis of secondary metabolites Biosynthesis of antibiotics	Amino acid biosynthesis and metabolism
*lld*D [PA4771]	Catalyzes the conversion of L-lactate to pyruvate. Is coupled to the respiratory chain.	Lactate oxidation Lactate metabolic process	L-lactate dehydrogenase activity FMN binding Oxidoreductase activity	Metabolic pathways Pyruvate metabolism Heterolactic fermentation	

**TABLE 2:** Pairwise score of interacting genes in the studied network including genes from GWAS output and their closely linked predicted functional neighbors as shown by STITCH5 database.

Interacting genes/metabolites		Combined interaction score
*cls [PA5394]*	*cds*A [PA3651]	0.986
*cds*A [PA3651]	*pur*A *[PA4938]*	0.523
*cds*A [PA3651]	*pur*D *[PA4855]*	0.442
*cds*A *[PA3651]*	*gua*A *[PA3769]*	0.554
*pur*D *[PA4855]*	Glutamine	0.879
*pur*D *[PA4855]*	GLutamate	0.885
*gua*A *[PA3769]*	GLutamate	0.985
*gua*A *[PA3769]*	Glutamine	0.985
*arg*J *[PA4402]*	*pur*A *[PA4938]*	0.813
*arg*J *[PA4402]*	*pur*D *[PA4855]*	0.449
PA0531 *[PA0531]*	*pur*A *[PA4938]*	0.565
PA0531 *[PA0531]*	*pur*D *[PA4855]*	0.551
PA0531 *[PA0531]*	*gua*A *[PA3769]*	0.400
PA0531 *[PA0531]*	*pur*N *[PA0944]*	0.443
PA0531 *[PA0531]*	Glutamine	0.892
PA0531 *[PA0531]*	GLutamate	0.869
*pur*N *[PA0944]*	Glutamine	0.879
*pur*N *[PA0944]*	Glutamate	0.885
*pur*N *[PA0944]*	*pur*D *[PA4855]*	0.999
*pur*N *[PA0944]*	*pur*A *[PA4938]*	0.682
*pur*N *[PA0944]*	*gua*A *[PA3769]*	0.859
*pur*N *[PA0944]*	O1O_08108 *[PA2444]*	Strong functional link 0.984
*pur*D *[PA4855]*	O1O_08108 *[PA2444]*	0.718
*pur*A *[PA4938]*	O1O_08108 *[PA2444]*	0.485
*gua*A *[PA3769]*	O1O_08108 *[PA2444]*	0.780
O1O_08108 *[PA2444]*	imm1 *[PA1151]*	0.518
O1O_08108 *[PA2444]*	PA0615	0.499
*pys*2 *[PA1150]*	imm1 *[PA1151]*	0.966
*pys*2 *[PA1150]*	PA0615	0.710
*pys*2 *[PA1150]*	PA1152	0.648
*pys*2 *[PA1150]*	PA1153	0.750
imm1 *[PA1151]*	PA0615	0.629
imm1 *[PA1151]*	PA1152	0.752
imm1 *[PA1151]*	PA1153	0.770
*gua*B [PA3770]	*pur*N *[PA0944]*	0.928
*gua*B [PA3770]	*pur*D *[PA4855]*	0.950
*gua*B [PA3770]	*pur*A *[PA4938]*	0.990
*gua*B [PA3770]	*gua*A *[PA3769]*	0.999
*gua*B [PA3770]	O1O_08108 *[PA2444]*	0.637
*gua*B [PA3770]	ClpV1[PA0090]	0.454
*gua*B [PA3770]	Glutamate	0.902
*gua*B [PA3770]	Glutamine	0.919
*clp*V1 *[PA0090]*	VgrG1[PA0091]	0.994
VgrG1[PA0091]	PA0082	0.973
XerC[PA5280]	PA0082	0.477
XerC[PA5280]	*lys*A [PA5277]	0.823
*lys*A [PA5277]	*gly*A1[PA5415]	0.742
*gly*A1[PA5415]	*gua*B [PA3770]	0.626
*gly*A1[PA5415]	*pur*N *[PA0944]*	0.988
*gly*A1[PA5415]	*pyk*A [PA4329]	0.423
*pyk*A [PA4329]	*lld*D [PA4771]	0.962

### 3.4 Effect of gentamycin-glutamate combination on permeabilization of the outer membrane

As a possible role of increased membrane permeabilization was proposed to be an underlying mechanism for increasing gentamycin susceptibility by glutamate, this was experimentally tested.

**FIGURE 7:**
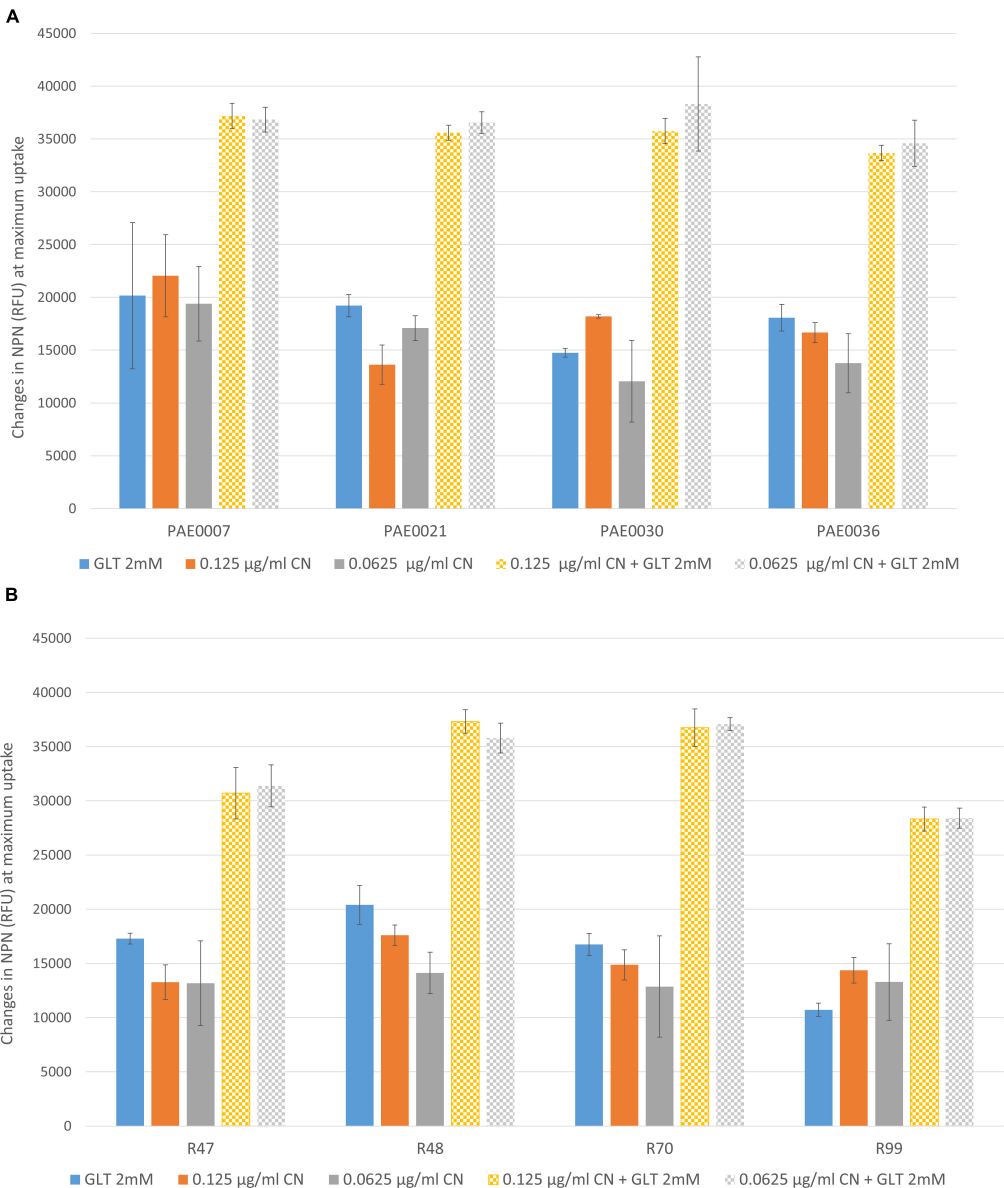
**(A,B)** Comparing the effect of glutamate/gentamycin separately and in combination as measured by change in relative fluorescence units. CN, gentamycin; GLT, glutamate; RFU, relative fluorescence units.

The uptake of fluorophore NPN in the presence of glutamate and two sub-inhibitory concentrations of gentamycin was measured by assessing the change in fluorescence at an excitation wavelength of 350 nm and an emission wavelength of 460 nm due to partition of normally impermeable hydrophobic NPN into bacterial membranes. Relative fluorescence values of replicates were determined by subtracting the fluorescence value of the tested isolate without the test compound (Results shown represent the maximum uptake values reached). Results are shown in Table 3 and Figure 7.

**TABLE 3:** Comparing outer membrane permeabilization by different test compounds.

Tested isolates	Relative NPN fluorescence (Mean ± SD)				
	GLT 2 mM	0.125 μg/ml CN	0.0625 μg/ml CN	0.125 μg/ml CN + GLT 2 mM	0.0625 μg/ml CN + GLT 2 mM
PAE0007	20,158 ± 6,924	22,051 ± 3,883	19,397 ± 3,530	37,185 ± 1,191	36,833 ± 1,175
PAE0021	19,217 ± 1,052	13,626 ± 1,853	17,088 ± 1,174	35,588 ± 724	36,551 ± 1,027
PAE0030	14,745 ± 405	18,205 ± 161	12,055 ± 3,855	35,742 ± 1,198	38,312 ± 4,455
PAE0036	18,066 ± 1,258	16,664 ± 956	13,763 ± 2,807	33,669 ± 725	34,595 ± 2,183
R47	17,285 ± 494	13,273 ± 1,586	13,183 ± 3,909	30,708 ± 2,374	31,382 ± 1,936
R48	20,399 ± 1,802	17,610 ± 937	14,133 ± 1,913	37,322 ± 1,090	35,796 ± 1,383
R70	16,745 ± 1,016	14,869 ± 1,385	12,868 ± 4,682	36,749 ± 1,730	37,083 ± 597
R99	10,720 ± 612	28,322 ± 1,110	28,412 ± 924	14,359 ± 1,183	13,288 ± 3,531

CN, gentamycin; GLT, glutamate.

## 4 Discussion

The significance of alterations in cell membrane permeability in aminoglycoside-resistant clinical isolates of *Pseudomonas aeruginosa* has been previously acknowledged (Kettner et al., 1995). Impermeability-related resistance has been linked to changes in outer membrane composition of *P. aeruginosa*, including alterations in the structure of lipopolysaccharide, overexpression of the outer membrane protein (OMP) OprH, and changes in the electron transport chain. Mutations associated with outer membrane porin changes (OprH-PhoP/PhoQ) have been linked to aminoglycosides resistance including resistance in clinical isolates (Shearer and Legakis, 1985; Yoneyama et al., 1991; Hasegawa et al., 1997). The synthesis of a faulty lipopolysaccharide (LPS) may hinder the absorption of aminoglycosides via the outer membrane (Bryan et al., 1984). Likewise, intricate quantitative or qualitative changes in the electron transport chain may hinder the active uptake of these drugs across the cytoplasmic membrane, restricting their intracellular accumulation to levels insufficient for ribosome inhibition (Taber et al., 1987).

Inactivation of *gal*U and *nuo*G genes was associated with impaired outer membrane uptake in addition to reduced active transport (El’Garch et al., 2007). The inactivation of some genes, including *nuo*G, *rpl*Y, and *gal*U, has shown previously to gradually increase AGs resistance by reducing proton motif force, modifying the AGs target, and impairing AGs binding and uptake in the laboratory strain, respectively (Dean and Goldberg, 2002). NuoG operon codes for proton-translocating type I NADH oxidoreductase. The inactivation of NADH dehydrogenase, an enzyme complex that plays a crucial role in establishing the proton electrochemical gradient, has been demonstrated to disrupt membrane energetics and thus hinder the uptake of aminoglycosides. The *gal*U gene product (UDP-glucose pyrophosphorylase) facilitates the transformation of glucose-1-phosphate into UDP-glucose, a crucial component for the assembly of a full LPS outer core. Previous research has demonstrated that *gal*U deletion leads to the synthesis of truncated (rough) LPS molecules devoid of both A- and B-band polysaccharides in *P. aeruginosa* (Dean and Goldberg, 2002). In addition, loss of the A- and B-band LPS was discovered to diminish the antibacterial efficacy of aminoglycosides by hindering their attachment to the cell surface (Kadurugamuwa et al., 1993).

Disruption of the genes; *pst*B (encoding a component of a high-affinity phosphate transport system), *fao*AB (encoding a multienzyme complex involved in degradative fatty acid oxidation) and *lpt*A encoding lysophosphatidic acid acyltransferase (LPA), responsible for adding the second FA to glycerol-3 phosphate in the synthesis of phospholipids (PLs) has shown to be associated with increased aminoglycosides susceptibility (Krahn et al., 2012). Mutations in *pts*B has been proposed to have a role in low-level tobramycin resistance (Sanz-García et al., 2018). Arn (PA3552-PA3559) LPS modification genes have also been linked to aminoglycosides resistance. The expression of the *arn*BCADTEF operon is recognized to restrict the interaction and self-mediated uptake of polycationic antibiotics (Schniederjans et al., 2017).

Being considered an important resistance mechanism and an essential step in aminoglycoside action, we have proposed that genetic variability of the genes involved in aminoglycosides uptake may underlie the observed phenotype of enhanced glutamate/aminoglycoside uptake and consequently its killing effect. However, when the tested isolates were analyzed through constructing a genetic relatedness phylogram based on these genes, the isolates did not show obvious clustering or separation. Although not extensively studied, we have also proposed that variability in genes involved in glutamate uptake and utilization in *P. aeruginosa* (Lundgren et al., 2021) may interpret such behavior of enhanced killing. However, the genetic relatedness phylogram based on glutamate uptake genes and combination of both glutamate and aminoglycoside uptake also did not show obvious clustering of isolates with different behavior and the genetic relatedness phylograms were not able to separate the two groups.

Analysis of gentamycin/glutamate uptake across the cell membrane demonstrated that the combination exhibited an increased uptake through bacterial membrane compared to each of the agents when separately used and this effect was similarly observed in all tested isolates. Increased outer membrane permeability may be attributed to either changes in outer membrane porins or to damage to the outer membrane or structural loosening due to Mg2+ removal facilitating the passage of bigger molecules across the cell membrane, leading to bacterial toxicity (Lam et al., 2014). The inactivation of exogenous carbon by aminoglycoside antibiotics has previously been linked to heightened NADH levels, increased proton motive force (PMF) production, and enhanced antibiotic uptake, however this has not been studied for glutamate. A second proposed mechanism for glutamate effect involves the integration of D-amino acids into microbial peptidoglycan, which alters the sequence of existing amino acids and substitutes the terminal D-alanine (at the fifth position), thereby increasing its sensitivity and susceptibility to antimicrobial agents (De Jonge et al., 2002). A third mechanism that may be involved is the potential of glutamate to act as glutamine synthetase inhibitors exerting an antimicrobial effect (Berlicki, 2008).

Based on that, we proposed that other unknown factors or mechanisms may underlie such behavior and in order to investigate that, a GWAS study was performed to investigate genes with probable role in such an effect. GWAS was performed to interpret the phenotypic behavior observed with enhanced glutamate/gentamycin uptake across bacterial membrane and enhanced killing effect of the combination on *P. aeruginosa*. The metabolic environment has been demonstrated to influence to efficacy of antibiotic mediated killing where inefficient metabolic flux has been linked to antibiotic resistance (Xiang et al., 2023). Metabolite-based metabolic reprogramming has been suggested as an effective approach to reverse antibiotic resistance, and we show evidence for that in the current study. The role of glutamate metabolism and purine metabolism in metabolic mediated antibiotic killing efficacy has been recently demonstrated (Xiang et al., 2023) where inactivation of glutamate metabolism and purine metabolism were linked to inactivated glycolysis, inactivated P cycle, and depressed glucose flux resulting in antibiotic resistance and this can be supported by the findings from the current study.

When GWAS was applied, genes involved in glycerophospholipid metabolism, purine metabolism, and glutamate metabolic pathways were interconnected and showed significant difference among the two phenotypic groups of tested isolates (Figure 6). Cardiolipin synthetase gene [PA5394] showed significant difference in the output of GWAS study. The gene from the metabolic pathways of glycerophospholipid metabolism and the super pathway of phospholipid biosynthesis I was linked to each of *pur*D [PA4855], *pur*A [PA4938], and *gua*A [PA3769] from the metabolic pathways of purine metabolism through *cds*A [PA3651] which belongs also to glycerophospholipid metabolism and the super pathway of phospholipid biosynthesis I. Each of *pur*A [PA4938] and *gua*A [PA3769] also belonged to the pathway of glutamate metabolism (Table 1). Interaction scores are shown in Table 2. The *pur*N [PA0944] gene belonging to the pathway pf purine metabolism also acted as a hub gene connecting each of cardiolipin synthetase gene [PA5394], *cds*A [PA3651], *pur*D [PA4855], *pur*A [PA4938], and *gua*A [PA3769] on one side and to the hub gene O1O_08108 [PA2444] from amino acid biosynthesis and metabolism pathways that connect all these genes to each of *pys*2 [PA1150] and the pyocin S2 immunity protein imm1 [PA1151] from the adaptation and protection pathways which also showed significant differences in GWAS output analysis performed in this study. Also, *lld*D [PA4771] showed significant differences in GWAS output analysis and was interconnected to genes involved in glutamate through each of *pyk*A [PA4329], *gly*A1[PA5415], and *gua*B [PA3770].

Many of the genes involved in these interconnected pathways appear to play a role in glutamate metabolism including *pur*A [PA4938], *gua*A [PA3769], *pur*D [PA4855], PA0531 and *pur*N [PA0944] pointing to the important role glutamate may play in these interactions. Analysis of gene network interactions demonstrate that glutamate metabolic pathways show functional links to membrane phospholipid synthesis and also links to nucleotide metabolism as evidenced by the interacting set of genes in Tables 1, 2, and Figure 6.

There is literature evidence showing that glutamate hyperproduction can occur in phospholipid deficient cytoplasmic membranes (Sanchez et al., 2017). A previous study has demonstrated that altered phospholipid composition induced by several genes including *cls* and *cds*A specifically affect L-glutamate efflux and propose that a specific lipid environment may be required to activate a presumed glutamate carrier (Nampoothiri et al., 2002). The current study points to similar findings about the link of membrane lipid composition to glutamate movement across the cell membrane where glutamate movement across the cell membrane is probably enhanced across the cell membrane and increasing the gentamycin uptake with a specific altered cell membrane phospholipid composition.

Recently, mechanosensitive channels have also been identified as exporters of l-glutamate in *Corynebacterium glutamicum* (Kawasaki and Martinac, 2020). It has been demonstrated that abundance of negatively charged lipid components, such as cardiolipin and phosphatidylglycerol, in the cytoplasmic membrane underly membrane softness which affects mechanical properties of the membrane lipid bilayer that consequently affect the gating mechanisms of mechanosensitive channels (Klatt et al., 2018). Mechanosensitive channels are activated by the force exerted by lipids, making it essential to comprehend the intricacies of the interactions between proteins and lipids to elucidate their gating mechanism (Kawasaki and Martinac, 2020). We propose that gentamycin-glutamate binding may induce ionic and membrane potential changes that may lead to conformational rearrangements in hook lipids gating such channels increasing uptake across membranes, however this should be experimentally tested.

We show that combination of glutamate with gentamycin experimentally increases uptake of gentamycin across the cell membrane and we propose based on GWAS results that altered membrane lipids may induce activation of certain channels that enhance membrane permeability to glutamate.

Interestingly observed is the possible involvement of pyocin S2 and pyocin S2 immunity protein adaptation and protection system in gentamycin-glutamate mediated bacterial killing. Results of GWAS show significant differences in these genes among the two phenotypic groups tested pointing to their possible activation and role in bacterial killing induced by excess glutamate uptake. Network analysis shows their relatively strong linkage to some hypothetical proteins with unknown functions that are linked to O1O_08108 [PA2444] (Table 2) which is strongly linked in turn to other genes involved in glutamate metabolic pathways including *pur*N [PA0944], *pur*D [PA4855], *pur*A [PA4938], and *gua*A [PA3769]. It is possible that excess glutamate uptake leads to activation of pyocin S2 which in turn enhance antibiotic mediated bacterial killing, however such hypothesis requires further experimental testing.

The *gua*B [PA3770] gene connected *vgr*G1[PA0091] to each of the previous networks through interaction with each of *clp*V1[PA0090], O1O_08108 [PA2444], *pur*N [PA0944], *pur*D [PA4855], *pur*A [PA4938], and *gua*A [PA3769] (Table 2), thus connecting the pathways of nucleotide biosynthesis and metabolism, fatty acid and phospholipid metabolism, and glutamate metabolism to bacterial secretion system. Interestingly observed that *xer*C [PA5280] showed significant difference among the two tested phenotypic groups. In addition to being linked to many interacting genes in the studied network, *xer*C was directly linked to *vgr*G1[PA0091] through the hypothetical protein PA0082 in the studied network and also linked to the hub gene *pur*N [PA0944] through the interacting genes; *gua*B [PA3770], *lys*A [PA5277], and *gly*A1[PA5415] (Table 2). Members of the λ integrase family of site-specific recombinases such as *xer*C have been described to promote conservative reciprocal recombination and DNA rearrangements in *E. coli* and *P. fluorescens* (Dekkers et al., 1998). This rearrangement was linked to phenotypic switching regulating the expression of phase-variable cell surface antigens including surface lipoprotein, LPS, and fimbriae (Dybvig, 1993).

Being among genes showing significant difference among the two tested phenotypes and being functionally related to other GWAS significant output genes including *vgr*G1[PA0091] and *cls* [PA5394] raise the possibility of regulating certain DNA rearrangement that may produce different forms of LPS or induce variations in surface lipoproteins that may underly phenotypic switching or phase variation leading to enhanced glutamate-gentamycin uptake. This forms a hypothesized target that requires further experimental testing. Additionally, *lld*D [PA4771] was among the top genes showing differences in the GWAS study. The *lld*D is among the genes responsible for lactate utilization as an energy source by *Ps. aeruginosa* (Wang et al., 2018) and mutants in this gene showed defective growth in media containing l-lactate as the sole carbon source. Membrane phospholipids showed activation of L-Lactate Dehydrogenase from Membranes of *Escherichia* (Kimura and Futai, 1978) pointing to a possible interaction effect of membrane phospholipid and *lld*D in *P. aeruginosa* affecting different aspect of lactate and glutamate metabolism.

Aminoglycosides including gentamycin are proposed to cross the outer cell membranes via self-promoted uptake that involve disruption of the lipopolysaccharides (LPS)-Mg+ cross bridges (Krause et al., 2016). Aminoglycosides, being polycationic in nature, share the same binding sites of the naturally stabilizing cation Mg+ cross bridges. It is proposed that aminoglycosides exert its action by displacement of Mg+ cross bridges which leads to localized destabilization or distortion of the LPS membranes. The observed effect of glutamate on modifying gentamycin resistance can also be hypothetically interpreted in different ways. First, glutamate can act as an alternative electron acceptor during gentamycin cytoplasmic membrane transport in the energy dependent phase of gentamycin uptake. Similar research observations show that alternate terminal electron acceptors can promote electron transport and AGS uptake and killing. Second, glutamate can act by permeabilizing the outer membrane through phase I ionic binding through simple electrostatic interaction by chelation or displacement of Mg+ cross bridges exposing more binding sites for aminoglycosides and consequently increasing its uptake into the cell. Lastly, glutamate can also modify the sensitivity of gentamycin through post-uptake effect on glutamate cellular metabolism. However, all these proposed hypotheses require further experimental testing.

## 5 Conclusion

All the data presented point to the possible role of glutamate metabolism in modifying the effect of gentamycin on bacterial cell survival. Research data shown here suggests that this role can occur either due to its adjuvant effect on cell membrane permeabilization, because of certain changes in glutamate uptake and metabolic pathways or both together.

Data presented in this research point to the promising enhanced antibacterial effect of gentamycin and glutamate combination which is probably related to increased cell membrane permeability or to glutamate metabolic pathways. An important clinical application of the current research findings is the possible use of the combination of gentamycin and glutamate allowing gentamycin use at lower doses more effectively which can consequently reduce the known nephrotoxicity and ototoxicity associated with gentamycin. Using the combination (glutamate/gentamycin) would offer a better alternative to using cell wall inhibitors in combination with gentamycin. This can be especially beneficial with topical applications such as in treatment of skin infections in burn patients and as topical eye drops.

Uptake of aminoglycosides through bacterial cell membranes is considered a crucial but not a completely understood step in the action of aminoglycosides. Metabolic natural alternative approaches that target cell membrane permeability changes can be considered a very promising new alternative treatment strategy.

## Data Availability

The datasets presented in this study can be found in online repositories. The names of the repository/repositories and accession number(s) can be found in this article/Supplementary material. Read files for Sequenced isolates used in the current study are submitted to the ENA (European 611 Nucleotide Archive) with study accession number: PRJEB31646.

## Supplementary material

Supplementary Table 1 GWAS output genes showing significant differences among the two comparison groups.
